# Flamingos use their L-shaped beak and morphing feet to induce vortical traps for prey capture

**DOI:** 10.1073/pnas.2503495122

**Published:** 2025-05-12

**Authors:** Victor M. Ortega-Jimenez, Tien Yee, Pankaj Rohilla, Benjamin Seleb, Jake Belair, Saad Bhamla

**Affiliations:** ^a^Department of Integrative Biology, University of California, Berkeley, CA 94720; ^b^School of Biology and Ecology, University of Maine, Orono, ME 04469; ^c^School of Chemical and Biomolecular Engineering, Georgia Institute of Technology, Atlanta, GA 30318; ^d^Department of Civil and Environmental Engineering, Kennesaw State University, Marietta, GA 30060; ^e^Interdisciplinary Graduate Program in Quantitative Biosciences, Georgia Institute of Technology, Atlanta, GA 30332; ^f^Nashville Zoo, Nashville, TN 37211

**Keywords:** birds, filter feeding, hydrodynamics, vortices

## Abstract

Flamingos feature one of the most sophisticated filter-feeding systems among birds, characterized by upside-down feeding, comb-like lamellae, and a piston-like tongue. However, the hydrodynamic functions of their L-shaped chattering beak, S-curved neck, and distinct behaviors such as stomping and feeding against the flow remain a mystery. Combining live flamingo experiments with live brine shrimp and passive particles, bioinspired physical models, and 3D CFD simulations, we show that flamingos generate self-induced vortical traps using their heads, beaks, and feet to capture agile planktonic prey in harsh fluid environments. When retracting their heads rapidly (~40 cm/s), flamingos generate tornado-like vortices that stir up and upwell bottom sediments and live shrimp aided by their L-shaped beak. Remarkably, they also induce directional flows (~7 cm/s) through asymmetric beak chattering underwater (~12 Hz). Their morphing feet create horizontal eddies during stomping, lifting, and concentrating sediments and brine shrimp, while trapping fast-swimming invertebrates, as confirmed by using a 3D-printed morphing foot model. During interfacial skimming, flamingos produce a vortical recirculation zone at the beak’s tip, aiding in prey capture. Experiments using a flamingo-inspired particle collection system indicate that beak chattering improves capture rates by ~7×. These findings offer design principles for bioinspired particle collection systems that may be applied to remove pollutants and harmful microorganisms from water bodies.

## Significance

Flamingos employ their feet, L-shaped beak, and head movements to induce directional flow and recirculating eddies, effectively entrapping agile planktonic prey, such as brine shrimp, in muddy and hypersaline waters. This study reveals that flamingos, far from being passive filter-feeders, are active predators that use flow-induced traps to capture agile invertebrates.

Flamingos are renowned for their morphological adaptations that enable them to filter-feed on microscopic particulate food and highly mobile planktonic crustaceans, such as copepods and brine shrimps, in muddy, highly alkaline, and hypersaline waters (1–6). Curiously, current knowledge about flamingos’ feeding behavior remains largely descriptive, focusing on its upside-down posture (1), the piston-like function of their enlarged tongue that pumps water through their beak (2), and particle retention by the comb-like lamellae along their beak (2). This perspective, however, does not include their active predatory strategies and the interaction between their morphological traits and the fluid environment. Furthermore, the roles of their L-shaped and chattering beak, elongated neck, and morphing webbed feet could reveal distinct mechanisms for filtering and prey capture.

To address these open questions, we combine experiments, including live animals, PIV analysis, 3D-printed physical models of beaks and feet interacting with live brine shrimp and sediments, and 3D CFD simulations. We demonstrate that flamingos generate directional and recirculating flows to entrap agile prey. They achieve this by asymmetric clapping of their mandibles, retracting their head and neck, and dynamic stomping with their morphing feet. The curved beak plays an instrumental role in skimming at the air–water interface. Our findings reveal flamingos as specialized predators that create vortical traps using their morphological adaptations to capture swarms of agile invertebrates. This work provides a functional hypothesis for the L-shape of the flamingo’s bill, rooted in unsteady flow dynamics.

## Tornado-Like Vortices from Head Retraction

We trained Chilean flamingos (*Phoenicopterus chilensis*) at the Nashville Zoo to feed from a water-filled aquarium over several weeks. Utilizing high-speed cameras and particle image velocimetry (PIV), we filmed and analyzed their feeding behavior, using fine food particles and oxygen bubbles for flow visualization (see *Materials and Methods* and *SI Appendix*, Figs. S1 and S2).

**Fig. 1.**
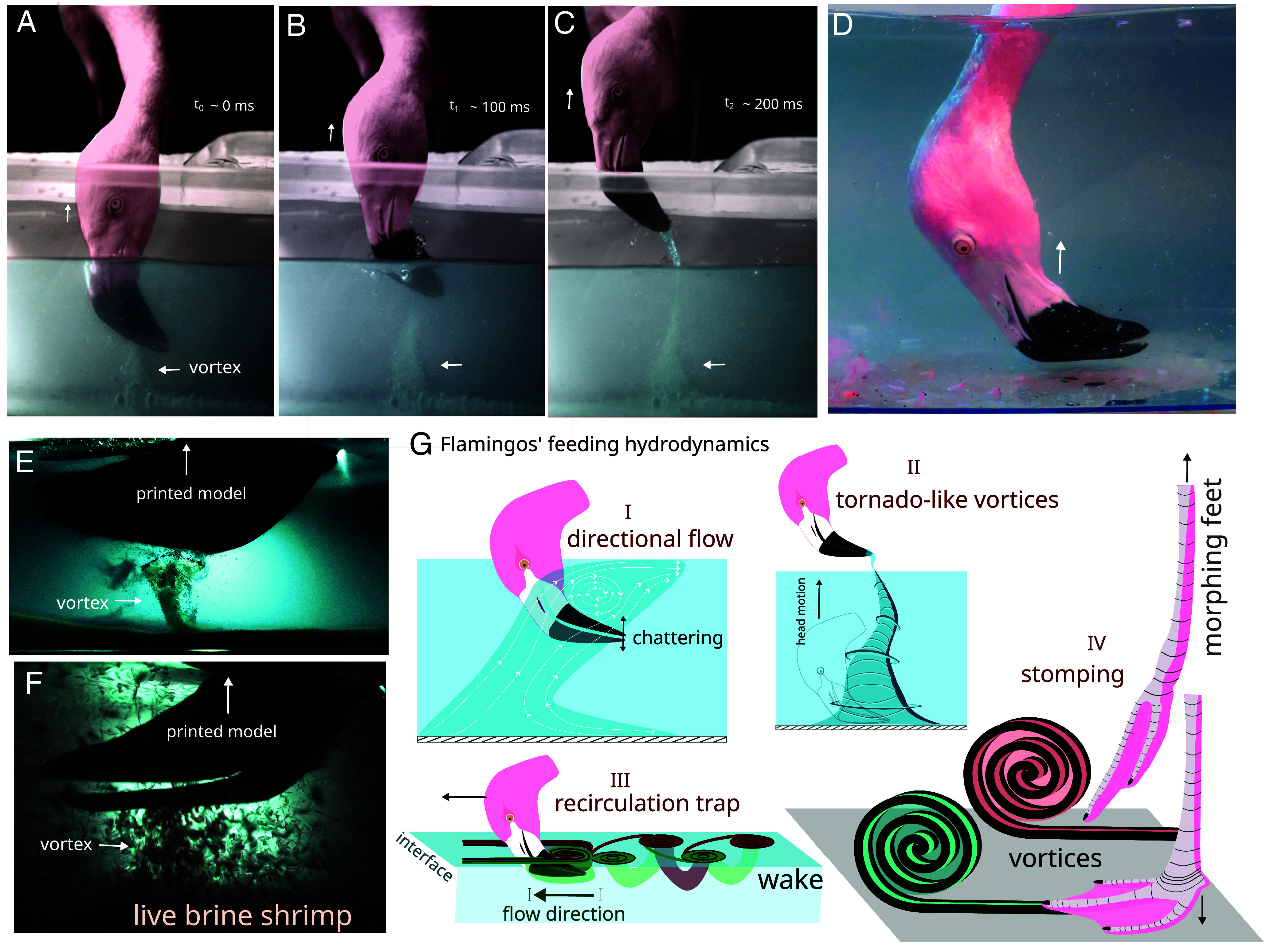
Vortices generated by flamingos. (*A*–*C*) Video frames that show a flamingo generating a tornado-like vortex when removing its head from the water. (*D*) Picture of a flamingo feeding at the bottom with its beak tip parallel to the horizontal. (*E*) Tornado-like vortex induced by a 3D-printed beak when pulled from the bottom. Horizontal arrow highlights the induced vortex. (*F*) Live brine shrimp entrapped within the induced tornado-like vortex. (*G*) Graphical representation of the flow structures produced by flamingos via beak’s chattering (*I*), head pulling (*II*), interfacial skimming (*III*), and feet stomping (*IV*). For details, see the main text.

While feeding upside down, flamingos frequently retract their heads from the bottom, facilitated by their elongated and S-curved flexible necks. This quick retraction (~40 cm/s), occurring in ~400 ms, produces strong tornado-like vortices, stirring particulate sediments at the bottom and upwelling them toward the surface (Fig. 1 *A*–*D* and Movie S1). During this upside-down feeding, the anatomical upper bill lies beneath and, due to the bent shape, presents a flat surface primed for vortical interaction.

In the laboratory, we 3D-printed a model of the flamingo beak to investigate this phenomenon further (*SI Appendix*, Fig. S3). We confirm that strong three-dimensional vortices are generated when the beak model is pulled from the bottom to the water’s surface (Fig. 1*E* and Movie S1). The L-shape of the beak, particularly the upper mandible resembling a flat plate, facilitates the generation of these vortices by creating a strong suction effect when pulled away from the fixed bottom surface of the aquarium. These tornado-like vortices trap and carry live brine shrimps and sediments to the interface (Fig. 1*F* and Movie S5).

## Directional Flow Generated by Asymmetric Beak Chattering

**Fig. 2.**
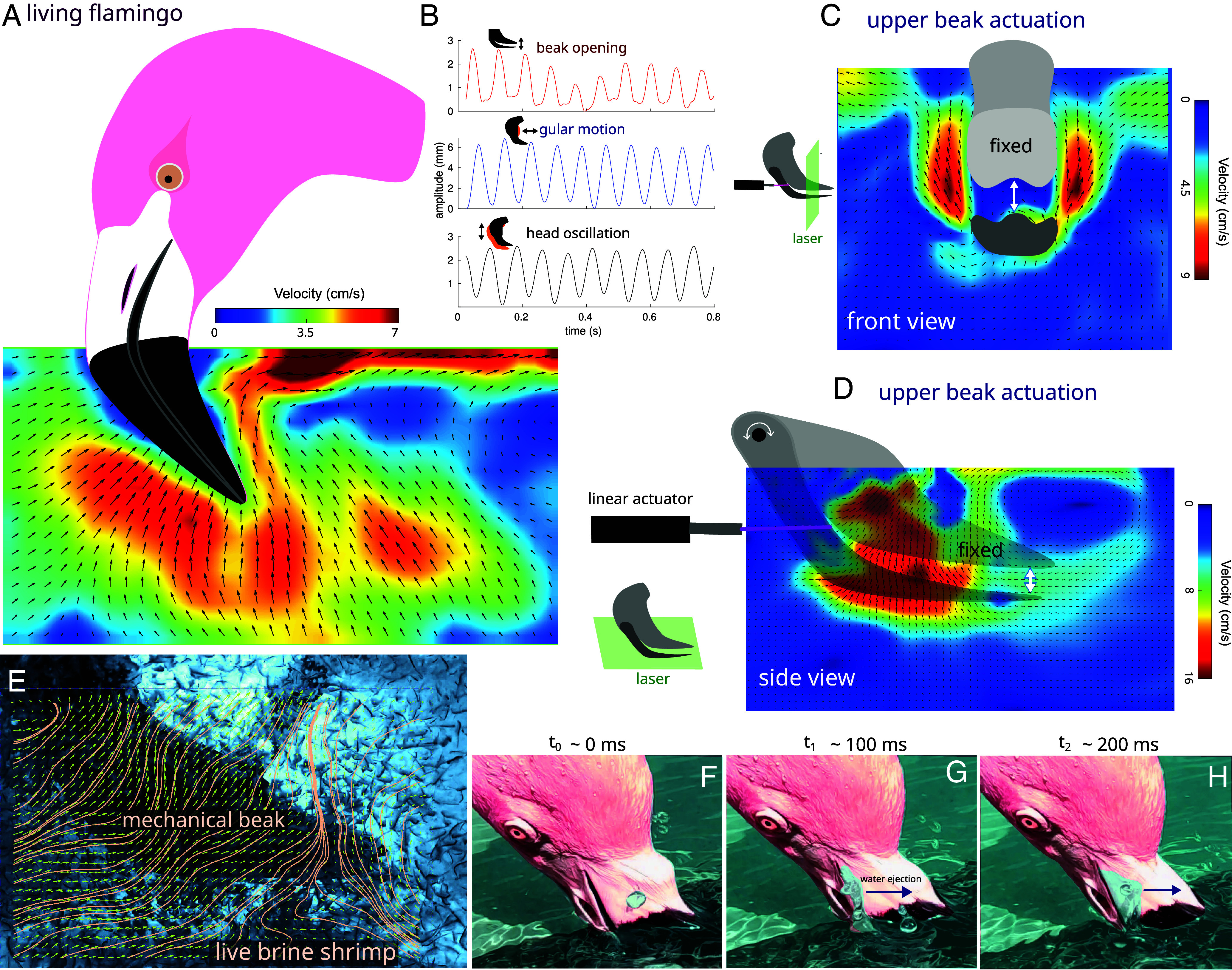
Directional flow by chattering. (*A*) Velocity field produced by a living flamingo during chattering. Notice the unidirectional flow induced by the beak. (*B*) Time series of the beak opening, gular motion, and head oscillation. (*C*) Front view of a velocity field analysis of mechanical oscillating mandibles. (*D*) Side view of a velocity field analysis of mechanical oscillating mandibles. Only the upper beak was actuated with a linear motor. Notice the unidirectional upward flow in *C* and *D* that agrees with that observed in living flamingos. (*E*) Streamlines showing the path followed by brine shrimp toward the beak (*F*–*H*). Flamingo feeding stationary at the surface of water and showing a fluid jet expelled along the beak.

We next investigated the beak opening-closing (chattering) dynamics in flamingos and found that during chattering, flamingos produce a vertical flow up to ~7 cm/s while “clapping” their mandibles at an oscillation rate of ~12 Hz (Fig. 2 *A* and *B* and Movie S2). This directional flow also occurs when flamingos feed while stationary at the water’s surface. Instead of expelling water outward, they expel a jet upward along the lower beak (Fig. 2 *F*–*H* and Movie S2), contrary to previous expectations (2). To understand how flamingos produce this directional flow, we used mandibles (donated by Zoo Atlanta) from a deceased Chilean flamingo (*P. chilensis*). We controlled the opening and closing of the specimen’s beak using a linear actuator attached to the upper mandible, while keeping the lower mandible fixed, replicating the behavior of live flamingos (Fig. 2 *C* and *D* and Movie S2). PIV analysis shows that the asymmetric clapping of ex vivo mandibles produces a directional flow consistent with that observed in live animals. To examine how chattering affects feeding behavior, we repeated the experiment using live brine shrimp (*Artemia sp.*). Our results show that *Artemia* cannot escape the flow generated by the chattering mandibles; instead, the flow carries them directly toward the beak (Fig. 2*E* and Movie S5).

Together, our findings from live flamingos, mechanical experiments, and live prey indicate that the oscillatory motion of the upper mandible during chattering creates a differential flow rate inside and outside the beak. This flow gradient establishes a persistent, directional upward inflow from the water’s bottom to the beak that brine shrimp cannot overcome, effectively trapping prey within the current. By channeling planktonic animals, such as brine shrimp toward the surface, where flamingos primarily feed, this directional flow may enhance prey capture and contribute to the effectiveness of their filter-feeding strategy.

## Vortical Stirring Via Morphing Feet

**Fig. 3.**
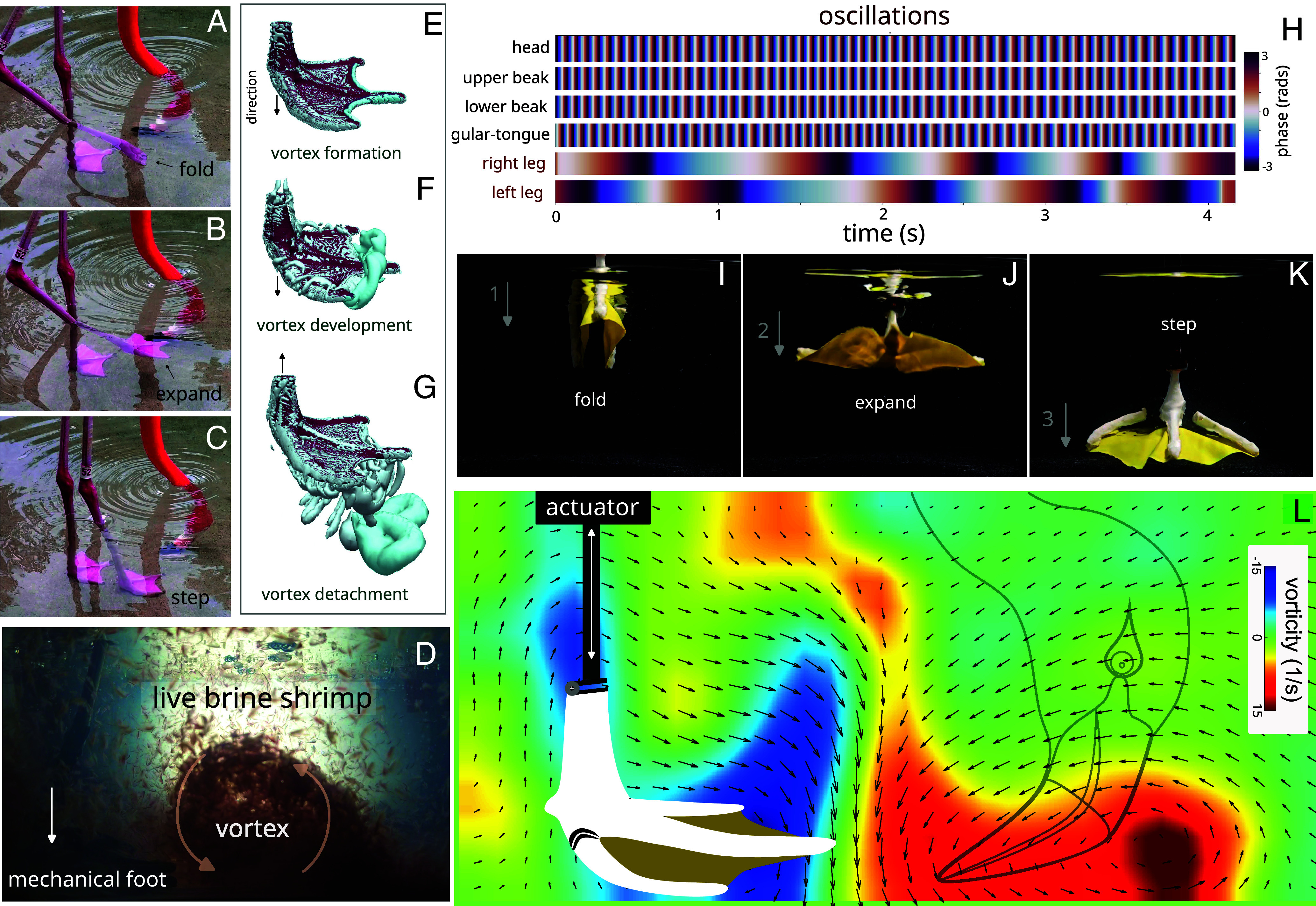
Flamingos’ hydrodynamics during stomping. (*A*–*C*) Video frames of a flamingo showing its right webbed foot moving upward and folded (*A*), downward and expanded (*B*), and finally when stepping on the bottom (*C*). (*D*) Video frame showing live brine shrimp entrapped by the vortex induced by the mechanical foot. (*E*–*G*) 3D computational simulation of the vortices produced by a reconstructed flamingo foot when moving downward (*E* and *F*) and upward (*G*). (*H*) Oscillation over time of the head, both mandibles, gular region, and both legs. (*I*–*K*) Engineered morphing foot that passively folds and expands, similar to a parachute. (*L*) Average vorticity field showing a strong vortex generated by the engineered foot during stomping. See details in the text.

Flamingos frequently stomp their feet in shallow water while positioning their heads upside down in front of their feet (Fig. 3 *A*–*C* and Movie S3). During each stomping cycle, a webbed foot spreads as it moves downward and folds as it moves upward. To examine the hydrodynamic role of foot stomping in feeding, we engineered a physical morphing foot model (*SI Appendix*, Figs. S3 and S4).

This model, built using a 3D-printed flamingo foot (toes and tarsometatarsus) and latex (webbed region), features three hinges that allow the lateral toes and foot to rotate. A connector secures the leg to a linear actuator, enabling controlled movement. The foot passively opens during the descending motion and closes during the upward motion, mimicking its live counterpart (Fig. 3 *I*–*K* and Movie S3). PIV measurements reveal that the morphing foot model produces a strong horizontal vortex with each cycle, reinvigorating the previously shed vortex (Fig. 3*L*). Experiments with live prey demonstrate that foot-stomping vortices can trap millimeter-scale aquatic organisms, from small copepods (1 to 2 mm) to larger species up to ~10 mm, including brine shrimp, mayfly larvae, and boatmen bugs (Fig. 3*D* and Movies S3 and S5).

We further corroborated these experiments with 3D multiphase computational fluid dynamics (CFD) simulations on a nonmorphing foot model moving in quiescent fluid (Fig. 3 *E*–*G*). These simulations reveal that the downward motion of the foot generates a pair of toroidal vortices. Flow structures detach from the front of the foot at the beginning of the upward motion, with the middle toe contributing to vortex generation. The asymmetry in toe and web morphology suggests that during foot stomping, vortices generated by both feet can travel inward and to the front, creating a strong vorticity region where the beak filter feeds. Simulations also show that the foot generates counterrotating vortices during upward and downward motions, consistent with experiments using a physical model of a nonmorphing foot (*SI Appendix*, Fig. S6 and Movie S3). Fig. 3*H* shows the oscillations of flamingo’s feet, mandibles, gular region, and head during stomping. These findings suggest that flamingos’ morphing feet generate coherent and strong vortical structures that help to entrap small prey (Fig. 3*D*), thus, boosting food harvesting while their heads are submerged upside down in the water in front of their feet.

## Kármán Vortices from Bent-Beak Skimming

**Fig. 4.**
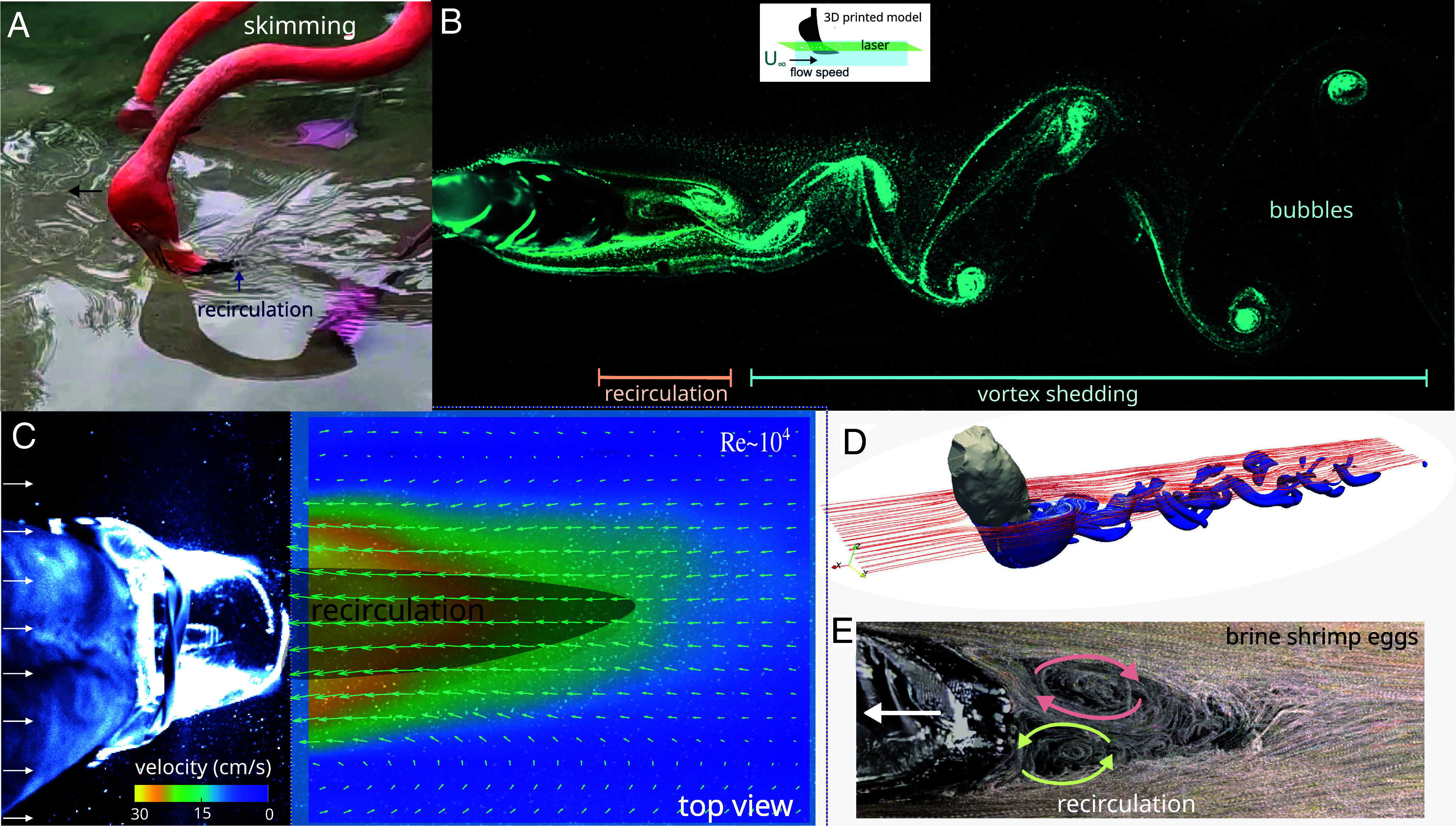
Skimming enhances particle and prey entrapment. (*A*) A flamingo feeding at the water surface. Notice that the beak’s tip is pointing downstream and in the same direction of the relative flow. (*B*) Flow visualization of the vortex shedding produced by a 3D-printed head. Notice the recirculation zone (collecting region) is located along the beak’s tip. (*C*) Average velocity field of a 3D-printed head in a water flume (Re ~ 10^4^). U_∞_ was subtracted from the vector field. (*D*) 3D computational simulation of wake produced by a flamingo head in a flow. (*E*) Brine shrimp eggs entrapped in the recirculation zone.

Flamingos exhibit an unusual feeding behavior at the water interface. In low-flow water bodies, flamingos position their submerged beaks parallel to the flow, with the tip of their beak pointing downstream (Fig. 4*A*). This is contrary to the typical behavior of any other filter-feeding vertebrates, such as whales and fish, which face the incoming flow during ram filter-feeding (7).

To investigate this apparent paradox, we placed a 3D-printed model of the flamingo’s head and beak in a flow tank, replicating their skimming orientation (Fig. 4*B* and Movie S4). PIV results reveal that the head model generates a von Kármán vortex street (Re ~ 10^4^), a series of counterrotating vortices commonly seen in cylinders placed in a flow stream (8). Additionally, a strong recirculation zone forms in the wake behind the head (vorticity ~30 s^−1^), where the flow reverses direction relative to the main flow (*U*_∞_ ~ 30 cm/s) (Fig. 4*C* and *SI Appendix*, Fig. S8 and Movie S4). The bent shape of the flamingo’s mandibles positions the beak tip within this recirculating zone, aligning it with the main flow, which is further confirmed with 3D-CFD simulations (Fig. 4*D*). By using live brine shrimp, both adults and eggs, we confirmed that they are collected and unable to escape the strong recirculation zone induced downstream (Fig. 4*E* and Movie S5).

Our findings suggest that the L-shape morphology of the flamingo’s beak facilitates skim-feeding at the air–water interface, enabling them to capture food particles within the recirculation zone. In contrast, juvenile flamingos and birds with straight beaks might not benefit from this mechanism. These results underscore the specialized feeding adaptation of flamingos, where their unique multifunctional L-shaped beak enables collection rate both at the water surface and the pond bottom.

## Beak Chattering Enhances Particle Collection and Prey Capture

**Fig. 5.**
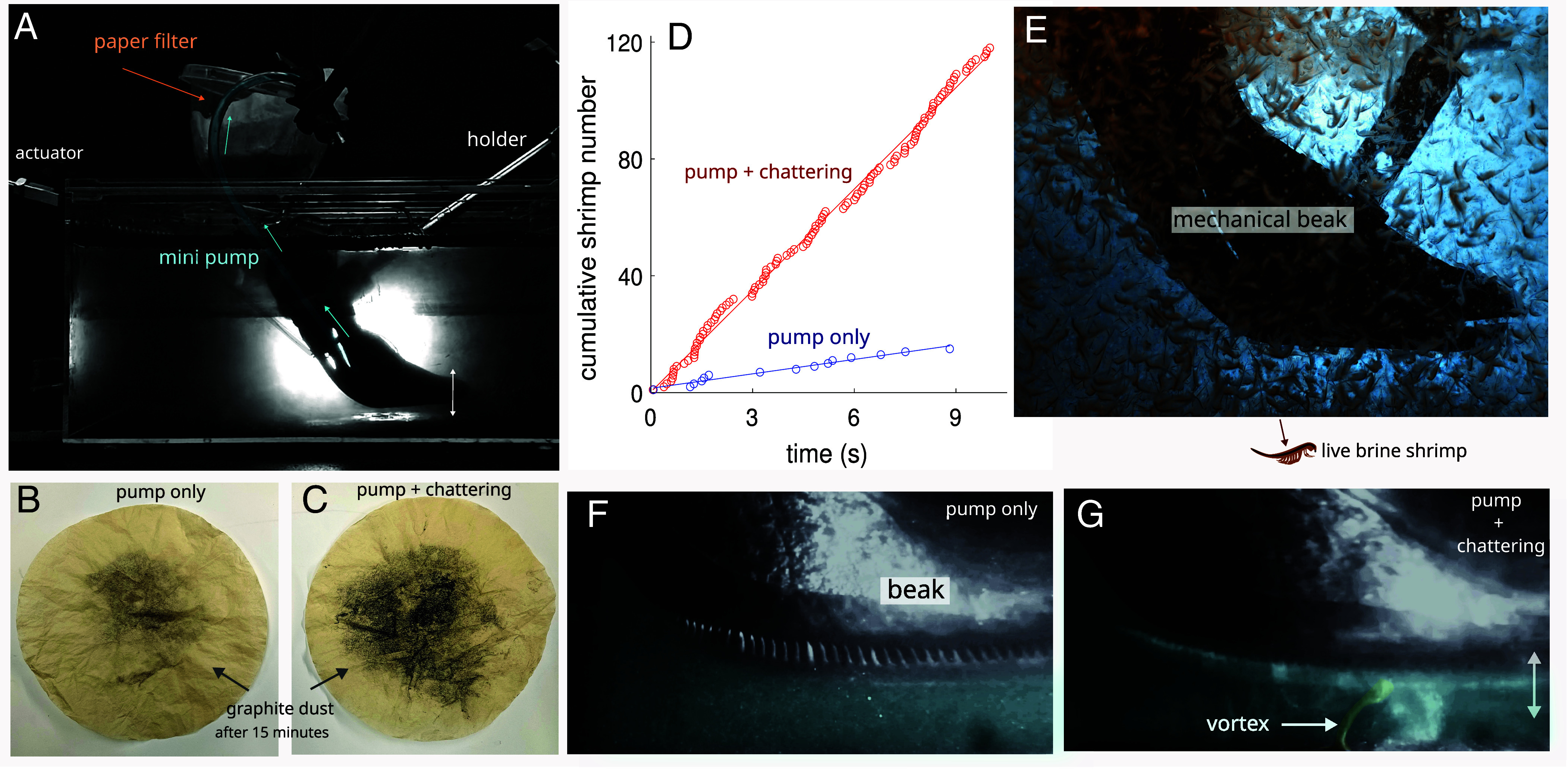
Role of chattering in flamingo feeding. (*A*) Particulate collection experiments showing the mechanical chattering mandibles and a mini pump that suctions water through a tube and pours it onto a paper filter that removes graphite particles added to the water. Graphite dust is filtered by coffee filters when only the pump is active (*B*) and when both the pump and the chattering mandibles are active (*C*). Notice the evidently larger dust amount filtered by the pump and the chattering beak acting together. (*D*) Cumulative shrimp captured when only the pump is active (blue, N_capture_=1.6 × Time + 1.5, R-squared = 0.97) and when both pump and chattering mandibles are active (red, N_capture_=11.6 × Time + 0.3, R-squared= 0.99). (*E*) Particle collection experiment using mechanical mandibles and live brine shrimp. Flow visualization showing a video frame when only the pump is active (*F*) and when both the pump and the chattering beak are acting together (*G*). Notice the mini tornado-like vortex induced by the chattering beak. See details in the text.

Finally, we evaluated the collection rate of the flamingo filter-feeding mechanism by combining an active pump with the chattering of the beak. Using a mini pump connected to a tube inside mechanical mandibles, we mimicked the water inflow generated by the piston-like motion of the tongue. The pump produced a flow rate of ~2 cm^3^/s in a small aquarium seeded with graphite dust to simulate sedimented food particles for flamingos. It has been estimated that flamingos can pump water five times faster than the rate we use in our experiments (9). As graphite particles settled to the bottom, they were recirculated and entrained in the flow, aided by the chattering motion of the mechanical mandibles. We operated the pump for 15 min, both with and without mandible chattering (Fig. 5*A*). To quantify the effect of chattering on feed uptake, we measured the number of particles collected by a paper filter placed at the pump’s tube outlet.

Our findings show that beak chattering increases the collection rate by ~9 times compared to trials using only the pump (Fig. 5 *B* and *C* and *SI Appendix*, Fig. S5). The clapping of the mechanical mandibles induces tornado-like vortices, lifting sediment particles toward the pump’s tube inlet (Fig. 5*G* and Movie S2). We then repeated the experiment using live brine shrimp instead of graphite particles. Chattering enhanced the capture rate of live brine shrimp by ~7 times, from ~1.6 to 11.6 shrimps per second, a trend qualitatively similar to the increased uptake of graphite particles (Fig. 5*D* and Movie S5). These results demonstrate that beak chattering significantly enhances the uptake of both suspended sediments and live prey such as brine shrimp.

## Discussion

Our results suggest that flamingos use their chattering mandibles, morphing feet, and S-curved long necks to self-generate vortical structures spanning from the bottom to the air–water interface. These vortices can entrap fast and agile prey, such as copepods and brine shrimps, as well as drifting food particles. Thus, flamingos actively create fluid dynamic traps to enhance prey capture, similar to specialized predators like spiders using webs, contrasting with the passive strategies of suspension feeders (10).

The unique L-shape of the flamingo’s beak has long been recognized as a major feeding adaptation, yet its hydrodynamic function remained a mystery (11). Our experiments indicate that the beaks’ shape helps induce vortical structures that aid feeding. Specifically, the curvature of the beak and flatness of the upper mandible create tornado-like vortices when the head retracts from the bottom. Additionally, during skimming, the beak’s shape aligns the tip with the flow, positioning it in the Kármán vortex wake’s collecting zone, allowing the animal to feed upside down and against the flow. Interestingly, right whales have similarly shaped filter feeding apparatuses (11). Recent evidence suggests that southern right whales may prey on small planktonic organisms at the seafloor (12), raising questions about whether these whales also induce vortices to trap and upwell prey through the interaction of their arched jaws with the seafloor.

Fossil evidence indicates that flamingos are distantly related to the Palaelodidae family, which had a straight bulky beak similar to the straight bills of juvenile flamingos during their first months after hatching (13). The oldest flamingo, *Harrisonavis croizeti*, had a less curved beak, appearing as an intermediate stage to the L-shaped beak of modern flamingos (14). Despite controversies about the feeding ecology of Palaelodids and ancient flamingos, the evolution from a straight to a bent beak likely increased their ability to capture smaller, active prey via hydrodynamic mechanisms. Juvenile flamingos, supplemented with esophageal secretions (crop milk) by their parents (15), may face limitations in capturing aquatic prey due to their straight bills. Studying the feeding strategies of juvenile flamingos is necessary to understand the ontogeny of beak development and prey capture.

The concept of self-induced vortical structures enhancing food intake is not unique to flamingos. Paddlefish (16), jellyfish (17), starfish (18), spoonbills (19), and phalaropes (20) also demonstrate this mechanism. For instance, phalaropes spin in circles on the surface of water using constant foot paddling to create tornado-like vortices that upwell and concentrate prey at the interface (21). This behavior allows phalaropes to effectively collect food with their pointy beaks. Interestingly, Wilson’s phalaropes can double their food intake by feeding near the water perturbations caused by flamingos during stomping (21). This highlights a potential mutual benefit where the vortices generated by flamingos can assist other species in prey capture. Shovelers, specialized filter-feeding ducks, also exhibit behaviors that might produce vortical structures to facilitate prey capture (22, 23). Their spoon-shaped beaks, covered with dense filtering lamellae, and their head movements, paddling, and circular swimming (in groups) likely contribute to this process (23). Comparative research on the fluid dynamics among filter-feeding birds, including flamingos and grebes, their closest relatives (24), is needed to understand how these birds use their adaptations for prey capture. Additionally, studying the interactions between multiple flamingos can show whether group stirring increases food intake, revealing possible cooperative vs. competitive flamingo hydrodynamic feeding strategies.

Stomping in shallow waters is common among both captive and wild flamingos (6), and linked to stirring and mixing sediments. Using a bioinspired morphing foot, we demonstrate that stomping creates recirculating vortices that lift particles and tiny organisms, concentrating them in front of the feet. Our mechanical morphing foot generates flow speeds up to 16 cm/s (*SI Appendix*, Fig. S7), trapping organisms with lower swimming speeds, such as copepods that move at ~0.1 cm/s (25). Even water boatmen bugs that can reach larger speeds of ~15 cm/s (26) experience reduced maneuverability and can be trapped in the eddies generated by the mechanical foot (Movie S3). Noticeably, flamingos commonly prey on brine shrimps that are abundant in hypersaline water environments (27). The maximal swimming speed that brine shrimp can reach is ~6 mm/s (28), which is two orders of magnitude less than the flow speeds induced by flamingos’ hydrodynamics. Our experiments with *Artemia* demonstrate that these tiny arthropods are unable to escape the directional flow and vortical traps generated by the oscillating mandibles, the head motion, and the morphing foot. Even at the interface, adult brine shrimps or their eggs were collected in the recirculation zone induced by a mechanical flamingo head while skimming at the interface. It is important to mention that brine shrimp eggs (i.e., cysts) are seen floating at the surface of salty lakes by the billions (29), thus, flamingos during skimming can trap and feed on these eggs through a Kármán vortex street. In addition, most water birds use their webbed feet to produce effective thrust underwater (30, 31), potentially facilitating sediment detachment near the bottom. In contrast, experiments with a rigid foot reveal that it produces vortices during upward motion, increasing the cost of lifting the legs and reducing the concentration of food particles in front of the foot (*SI Appendix*, Fig. S6 and Movie S3). Our study provides a foundation for future work on how aquatic birds use their webbed feet during stomping in shallow, muddy waters to generate vortical structures, offering insights into their feeding strategies and evolutionary adaptations.

Particle collection, filtration, and filter cleaning are major challenges in the industry due to clogging and fouling issues, especially on membranes (32). Hydrodynamic techniques such as hydrocyclones, pulsatile flows, and Taylor vortices have been developed to enhance membrane filtration (33). Engineers have also turned to fish-inspired cross-step filtration to reduce clogging (34). Previous descriptions of Chilean flamingo’s beaks indicate that they have marginal and submarginal lamellae distributed along both mandibles that together can filter particles as small as ~0.1 mm (27). Brine shrimp, a common prey of Chilean flamingos in South America (35) are one order of magnitude larger than that mesh size, which suggests that they can be easily trapped in the intermesh space. Our particle collection experiments with graphite dust and live *Artemia* demonstrate that chattering generates a directional flow toward the beak, enhancing the prey’s capture. Future experiments are needed to understand the flow dynamics inside the beak, induced by the deformable tongue and chattering beak, as well as the role of the lamellae to filter prey, for a better understanding of the flamingos’ filtering mechanism, including how clogging dynamics affects collection rates.

In conclusion, we found that flamingos actively generate vortical structures through beak oscillations, head retraction, foot stomping, and skimming to lift and concentrate prey and food sediments, improving their feeding performance in challenging environments. Understanding how flamingos use fluid dynamics to enhance particle collection could provide different perspectives on particle capture in biological and engineered systems.

## Materials and Methods

### Flow Visualization and PIV in Living Flamingos.

Chilean flamingos (*P. chilensis)* from the Nashville Zoo at Grassmere, Tennessee, were trained for several weeks to feed from a plastic tank (45 × 30 × 30 cm) filled with water. Shrimp and pellets were available ad libitum. After the training period, two flamingos were able to feed in the plastic tank. Experiment trials were performed in the off-exhibit corral for flamingos. For flow visualizations, we ground flamingos’ food pellets with a blender. Fine food particles (size ~3 mm) were poured into the tank and were allowed to settle down (*SI Appendix*, Fig. S2). We filmed flamingos that fed in the container using a FASTCAM Mini AX200 high-speed camera (Photron, Inc.) at 500 frames/s.

For particle image velocimetry measurements, we seeded the water with oxygen bubbles (size ~10 µm) using an oxygen emitter (O2 Grow 2040), as well as lycopodium particles (size ~30 µm), which are natural, nontoxic, and regularly consumed by humans as a nutritional supplement. We replaced the plastic tank with another made of clear plexiglass. A class-4 laser (Opto Engine LLC, 532 nm, 5 W) was used to produce a laser sheet illumination. We added frozen brine shrimp to encourage flamingos to feed in the plexiglass container. We filmed (1,000 frames/s) a side view of the flamingos feeding in the container (*SI Appendix*, Fig. S1). Recorded sequences were used to resolve velocity or vorticity fields, or both, with PIVlab (36) (https://www.pivlab.de/downloads). An interrogation window from 80 to 40 pixels, excluding those vectors with a SD greater than 5, was used.

### Mechanical Chattering Mandibles.

Both mandibles belonged to a deceased flamingo and were donated to us by the Zoo Atlanta. The lower mandible was fixed, while the upper mandible was actuated using a Mini DIY Design Reciprocating Cycle Linear Actuator with a DC Gear Motor (24 V, 5 to 1,000 RPM). The mechanical mandibles were placed inside a plexiglass container (30 × 260 × 15 cm) filled with water. Mandible oscillations were fixed to 8 Hz. We seeded the water with lycopodium particles. Side and front views were filmed. A FASTCAM Nova S6 high-speed camera (Photron, Inc.) was used for filming at 1000 frames/s. PIV analysis was as described previously.

### Filtering using a Pump and Chattering Mandibles.

To evaluate how the opening and closing of the beak influence filtering, we designed the following experiment. A water mini pump was connected to a tube (inner diameter 0.25 inches) inside the mechanical mandibles to induce flow suction inside the beak, mimicking the water inflow generated by the piston-like tongue (2). The volume flow rate generated by the pump was ~2 cm^3^/s. The water content in the aquarium used (30 × 20 × 15 cm) was seeded with graphite dust (~40 µm). We waited until most particles settled to the bottom. An outlet tube connected to the pump was placed outside the aquarium and poured the water on an unbleached coffee filter. The paper filter was used to retain the graphite particles coming from the water pump (Fig. 4*D*). We let the pump run for 15 min to collect the carbon particles in the coffee filter under two conditions: when both mechanical mandibles were still (Fig. 4*E*) and when they were chattering (Fig. 4*F*). We filmed at 250 frames/s in both treatments. To estimate the number of particles retained in the paper filter for each treatment, we simply used binary images from photographs to calculate the total number of black pixels (*SI Appendix*, Fig. S5). Then, we obtained the ratio of both resulting values.

### Physical Model of Flamingo Head and Tornado-Like Vortices.

A flamingo’s head was reconstructed using photogrammetry. Meshroom software (https://alicevision.org/#meshroom) was employed for the 3D reconstruction and MeshMixer (http://www.meshmixer.com/download.html) to correct and import the solid object to the 3D printer (Artillery Sidewinder X2). A thin plastic rod (15 cm) was glued to the base of the printed head (Fig. 1*E* and *SI Appendix*, Fig. S3) and used to orient and move the head manually. The head was placed gently at the bottom of an aquarium filled with water and with carbon particles sedimented at the bottom. Afterward, we manually pulled the head out of the water and filmed the retraction-induced vortices in water with a Photron FASTCAM Mini AX200 high-speed camera at 750 frames/s.

### Physical Model of Flamingo Head in a Flume.

Particle image velocimetry was performed on the wake produced by the 3D-printed flamingo head in a flow tank (Fig. 4*B*), mimicking the skimming behavior in flamingos. The tip of the beak was oriented downstream and parallel to the flow. The flow tank was seeded with oxygen bubbles using the oxygen emitter. The laser sheet was oriented horizontally, and the FASTCAM Mini AX200 high-speed camera was installed at the top of the tank to film at 1,000 frames/s. PIV analysis was done as we explained before. The flow speed of ~30 cm/s was selected based on previous study of Chilean flamingos wading in shallow waters (37). Thus, Reynolds number was ~7 × 10^3^.

### Mechanical Morphing Foot.

In the laboratory, we designed a bioinspired morphing webbed foot using a 3D-printed reconstruction of a flamingo’s foot through photogrammetry as previously described. Our physical foot model has hinges in their toes’ roots and in the tarsometatarsus region to allow bending (*SI Appendix*, Fig. S4). Flexible Latex was used as a webbed surface. We attached the engineered foot to a linear actuator (JQDML Reciprocating Linear Actuator 24V) to control the stomping frequency (~1.2 Hz). This mechanical foot can passively spread in the water (as a parachute) when moving downward, but it collapses when moving upward (Movie S3). Foot stomping was filmed with a high-speed camera Nova S6 (Photron Inc.) at 1,000 frames/s. Particle image velocimetry analysis of the mechanical foot during stomping was performed as described earlier.

### CFD.

Two computational fluid dynamic models were used for this study. The k-omega SST turbulence and Reynolds averaged Navier–Stokes (RANS) models were used in a finite volume method-based open-source library OpenFOAM (38). The pimpleFoam (39) and pimpleDyMFoam (40) solvers were chosen to simulate beak skimming and foot stomping, respectively. Solvers use the PIMPLE algorithm which can simulate transient incompressible fluid flows with the allowance of a large time stepping (41). To simulate the flamingo’s head in a flow stream, the rigid lid boundary condition was used for the water surface to save on computational costs; thus, surface tension effects at the interface are not considered. To capture the structure of the vortices, meshes were placed more densely near the head. Approximately 17 million cells were used for this simulation. A constant inflow of 3.1 cm/s was defined at the inflow boundary, and a zero gradient pressure boundary condition was prescribed at the outflow boundary to avoid reflection back into the domain. The k-omega SST turbulence (41, 42) closure model was used for this simulation due to its wide applicability (43) for various flow conditions. The time step was set to 0.01 s to maintain a Courant number less than one. The Reynolds number associated with this simulation was ~10^3^.

Foot stomping simulation uses a different numerical solver due to two primary reasons. First, the movement of the foot upward and downward requires a set of dynamic mesh. Second, the simulation, which consists of a rectangular domain, uses an unsteady Reynolds-averaged Navier–Stokes (RANS) model coupled with a dynamic mesh for the feet. The solid model of the flamingo’s foot was placed such that it could oscillate in the vertical direction within the rectangular domain at a fixed amplitude and frequency. The bottom boundary for the domain was a no-slip boundary, while zero gradient boundaries were used for the sides. In the k-omega SST turbulence closure model, the surface was defined as the rigid-lid boundary (41, 42). The temporal step was 1 ×10 ^−6^ s. The total number of cells used in this simulation was ~4 million cells. The oscillation cycle of the foot was modeled by a period of 17.45 rad/s with an amplitude of 3 cm.

### Pond Animals.

Copepods, mayfly larvae, and boatman bugs were collected from a pond near the University of Maine, Orono (Movie S3). These organism were kept in a tank and used to test whether the mechanical foot can induce vortices and entrap them during stomping.

### Live Brine Shrimp.

Live brine shrimp (adults and eggs) were obtained from the Carolina Biological Supply Company. Adults were kept in saline water with a specific gravity of 1.024, temperature of 25 °C, and pH of ~8. Experiments with these live organisms, using mechanical mandibles (Movie S5) and morphing mechanical feet, were performed at UC Berkeley and followed as described before. Experiments regarding skimming at the interface were performed by moving the head horizontally (~15 cm/s), while the water was kept still. For the filtering experiment, we counted the number of brine shrimps passing through the outlet tube over time. We compare capture rates over time when the pump was only active and when both the pump and chattering mandibles were active.

## Supplementary Material

Appendix 01 (PDF)

Movie S1. **Tornado-like vortices and head retraction**. [00:14 s] Live flamingo generating tornado-like vortices. [00:34 s] 3D-printed beak inducing tornado-like vortices when pulled upward from the bottom.

Movie S2. **Directional flow and beak chattering**. [00:14 s] Directional flow produced by a live flamingo during beak chattering. [00:19 s] Flamingo ejecting a water jet along the beak. [00:25 s] Mechanical beak producing directional flow through chattering. [00:40 s] Filtering experiment using a mini pump and a mechanical chattering beak. [00:45 s] Vortices induced by the chattering mandibles and the pump. [00:52 s] No evident vortical structures observed when only the pump is active.

Movie S3. **Horizontal vortex and stomping**. [00:14 s] Live flamingos stomping while feeding. [00:37 s] Mechanical morphing foot in motion. [00:46 s] Mechanical morphing foot producing horizontal vortices. [00:54 s] Morphing foot entrapping pond organisms via induced vortices. [01:13 s] 3D computational simulation of vortices generated by a flamingo foot. [01:25 s] Rigid mechanical foot inducing vortices during upward and downward motion.

Movie S4. **Kármán vortex street and skimming**. [00:12 s] Live flamingo skimming at the water-air interface. [00:25 s] 3D-printed flamingo head generating Kármán vortices and a recirculation zone. [00:46 s] 3D computational simulation of vortices produced by a flamingo’s beak.

Movie S5. **Live brine shrimp (Artemia sp.) and flamingos’ vortical traps**. [00:20 s] Mechanical beak producing directional motion of live Artemia by chattering. [00:40 s] Capture collection experiment using a mini pump and a mechanical chattering beak. [01:00 s] 3D-printed beak inducing tornado-like vortices of live Artemia when pulled upward. [01:21 s] Morphing foot entrapping Artemia in an induced vortex. [01:35 s] Artemia collected in the recirculation generated by a printed head during skimming. [02:10 s] Adults and nauplii of brine shrimp.

## Data Availability

Data sets, scripts and 3D reconstructions that support the findings of this study are available in Dryad (44). All other data are included in the article and/or supporting information.
