# Supplementary material for: Flamingos use their L-shaped beak and morphing feet to induce vortical traps for prey capture
Source: Proc Natl Acad Sci U S A. 2025 May 12;122(21):e2503495122. doi: 10.1073/pnas.2503495122 (PMC12130884; doi:10.1073/pnas.2503495122)
Supplement: Supplementary file 1 — Appendix 01 (PDF) [file pnas.2503495122.sapp.pdf]

Supporting Information for
Flamingos use their L-shaped beak and morphing feet to induce vortical traps for prey capture

Victor M. Ortega-Jimenez, Tien Yee, Pankaj Rohilla, Benjamin Seleb, Jake Belair, Saad Bhamla

Corresponding Authors Victor M. Ortega-Jimenez and Saad Bhamla
Email: vortex@berkeley.edu (VMO-J). saadb@chbe.gatech.edu (SB)

This PDF file includes:

Figures S1 to S9
Legends for Movies S1 to S5

Other supporting materials for this manuscript include the following:

Movies S1 to S5

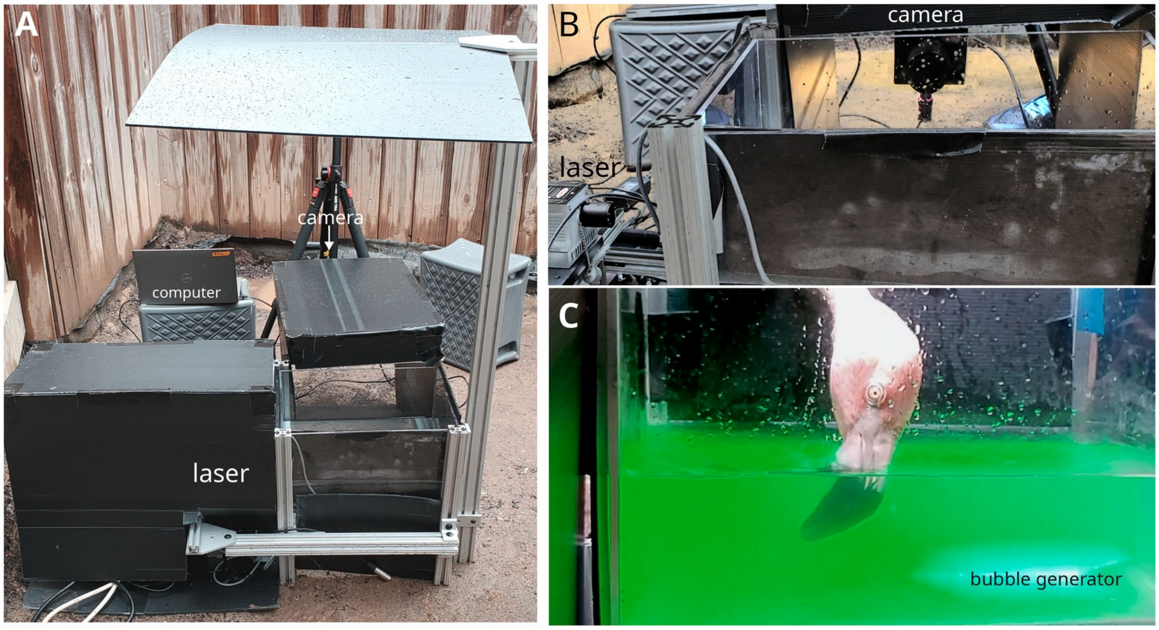

Fig. S1. PIV Setup. (A) Apparatus used to resolve the velocity and vorticity fields generated by feeding flamingos. **(B)** Front view of the apparatus, displaying the camera and laser. **(C)** Flamingo feeding in the apparatus, with the laser activated.

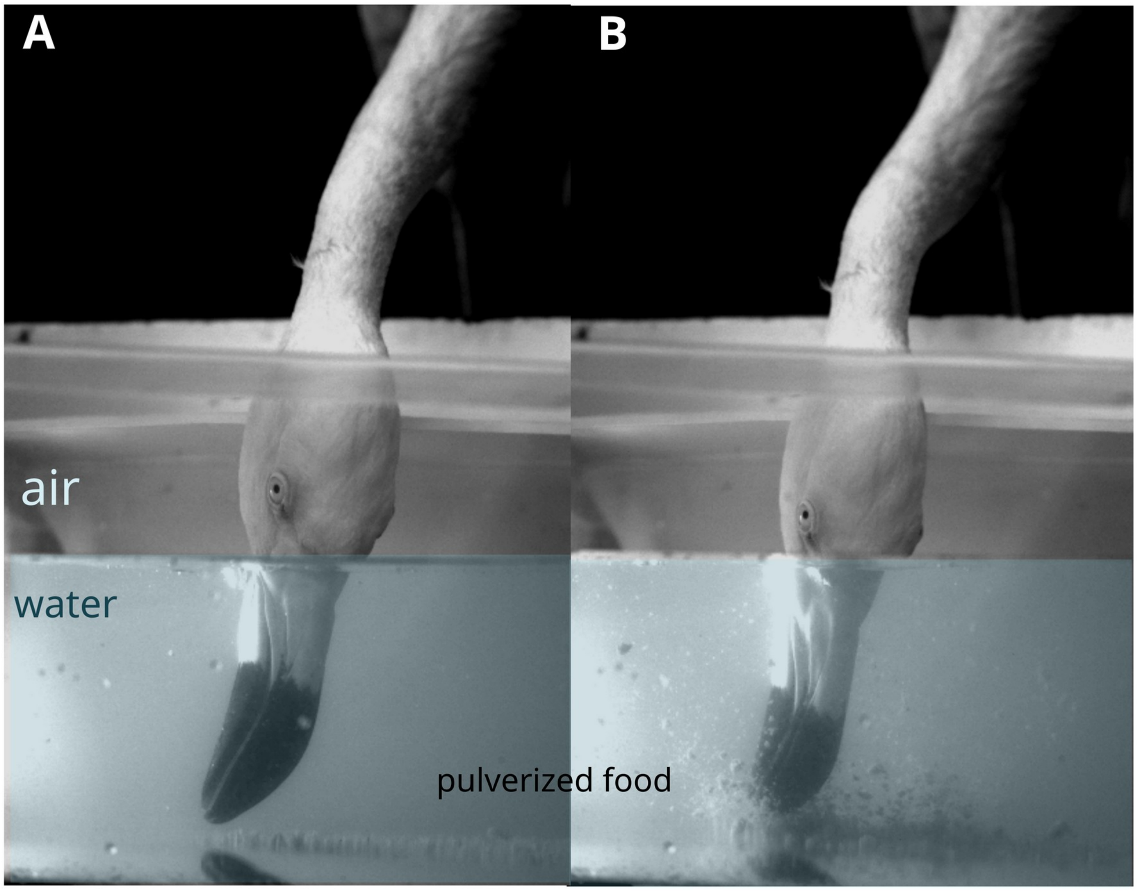

Fig. S2. Flow visualization using pulverized food. **(A)** Particles sedimented at the bottom of the container prior to the flamingo beginning to feed. **(B)** Flamingos stirring the particles during feeding.

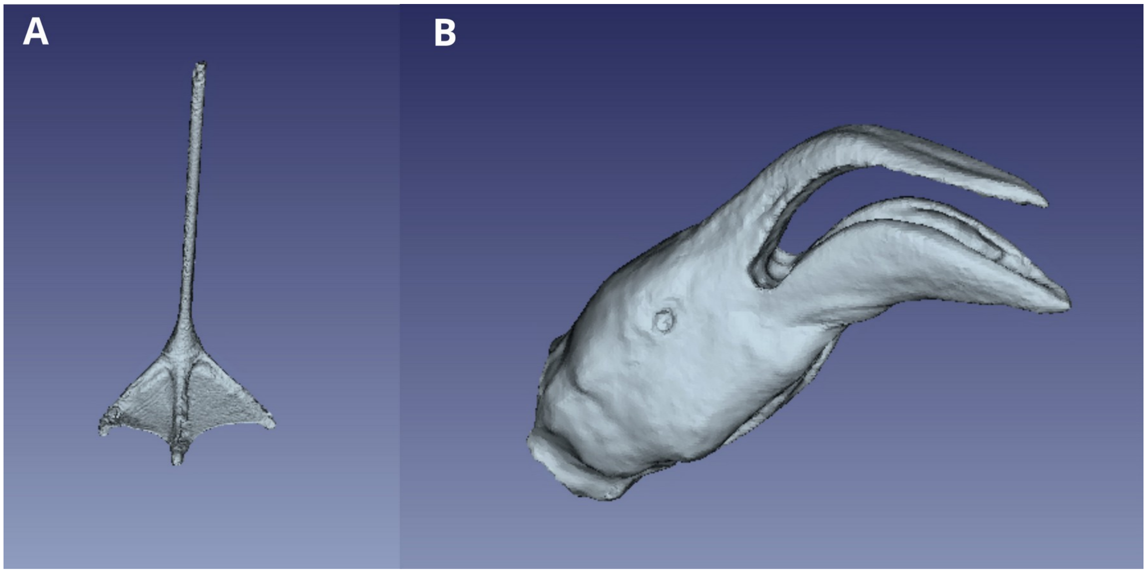

Fig. S3. 3D reconstruction using photogrammetry. **(A)** foot. **(B)** Head.

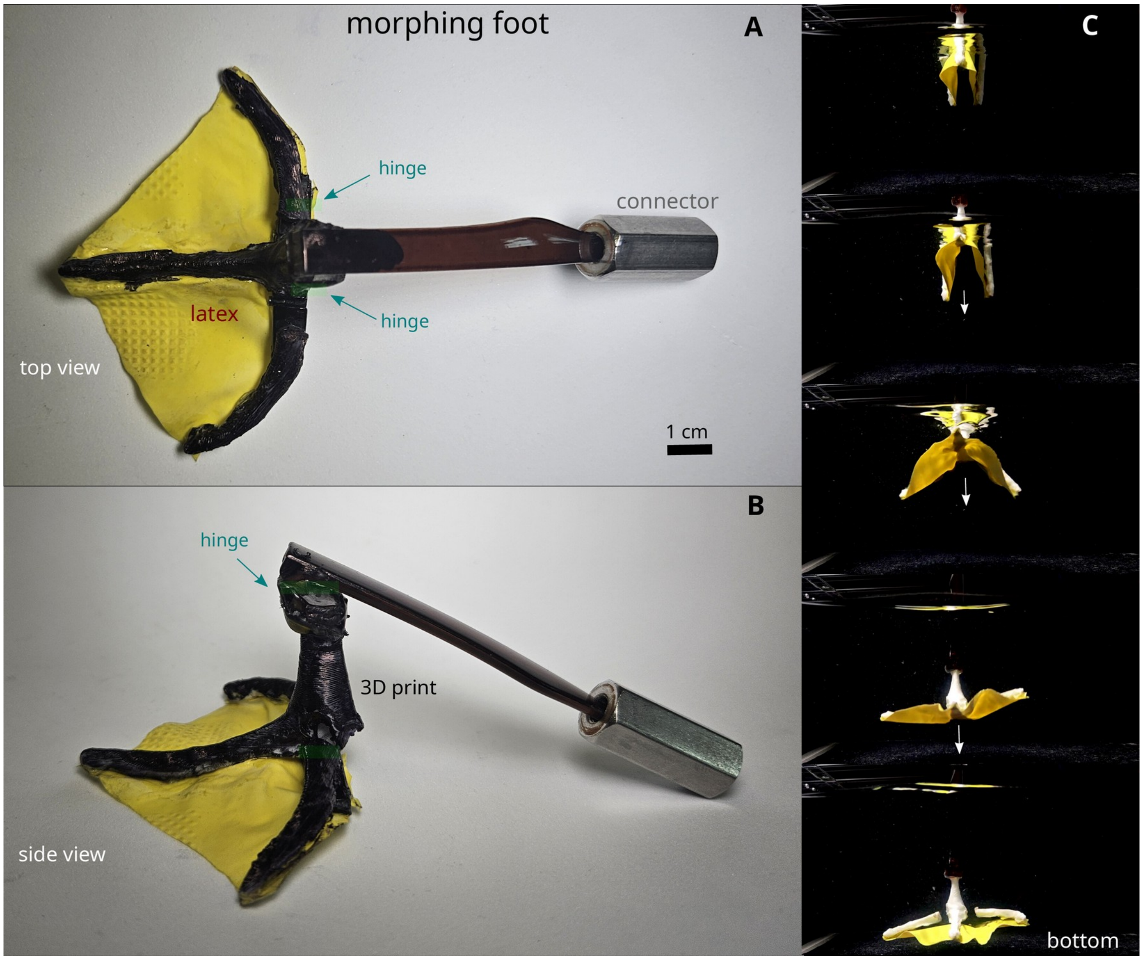

Fig. S4. Morphing foot design. **(A-B)** An engineered foot was created using a 3D-printed flamingo foot (toes and tarsometatarsus) and latex (webbed region). Three hinges facilitated the rotation of the lateral toes and foot. A connector was used to attach the leg to a linear actuator. **(C)**Video sequence showing the morphing foot during downward motion in water. Notice that the foot passively opens during descent and closes during upward motion (see movie S1).

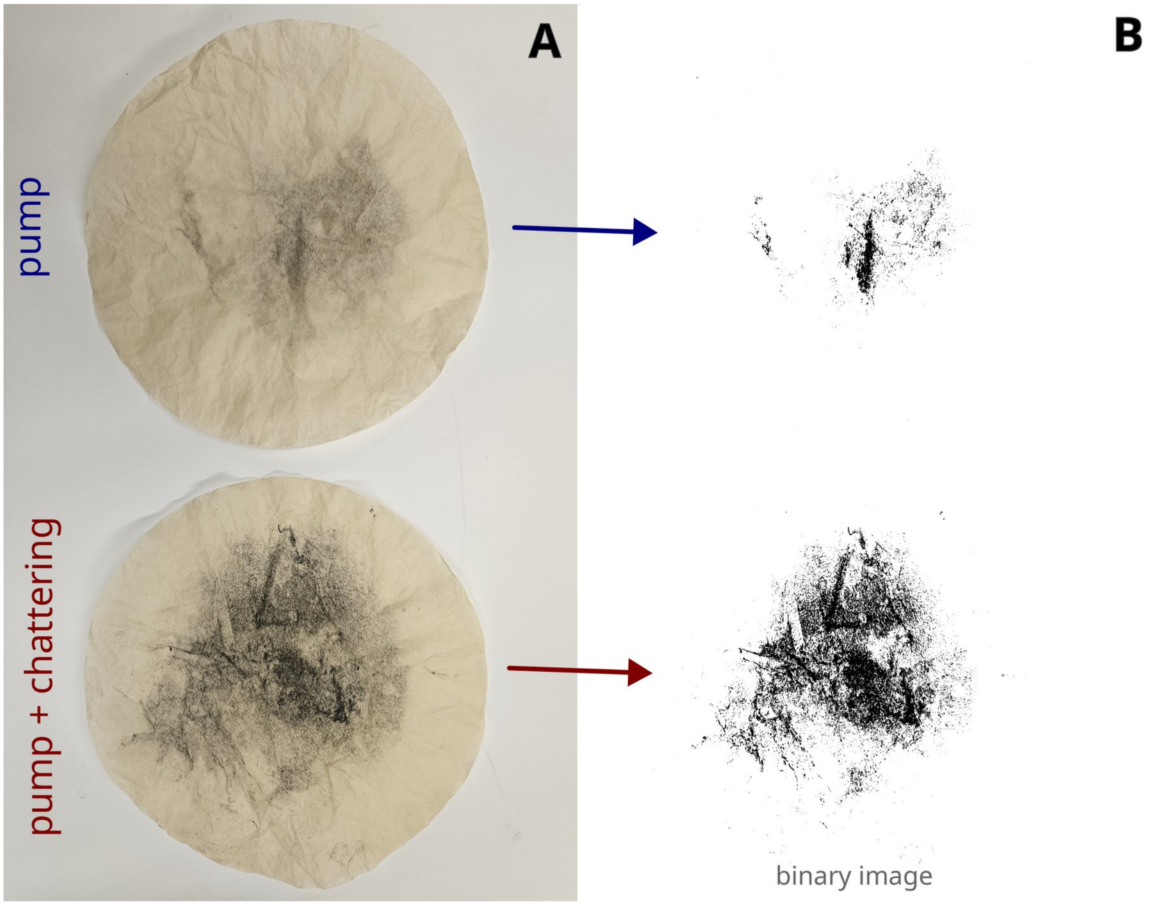

Fig. S5. Particle collection using a mechanical beak and a water pump. **(A)** Picture showing filter papers after using only a pump, and the pump combined with chattering. **(B)** Binary images of pictures in (A). The number of particles retained in each filter paper was estimated by calculating the total number of black pixels.

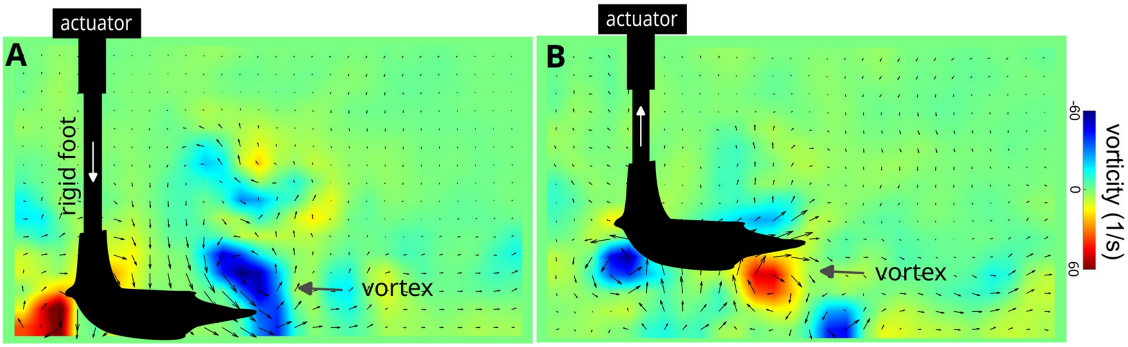

Fig. S6. Vorticity field of a rigid foot moving upward (A), and downward (B) at 1.9 Hz. Notice that vortices are induced during both downward and upward motion. This suggests that a rigid foot creates unnecessary drag during upward motion. In contrast, a morphing foot (see Fig. 3) does not produce a strong vortex or drag during upward movement.

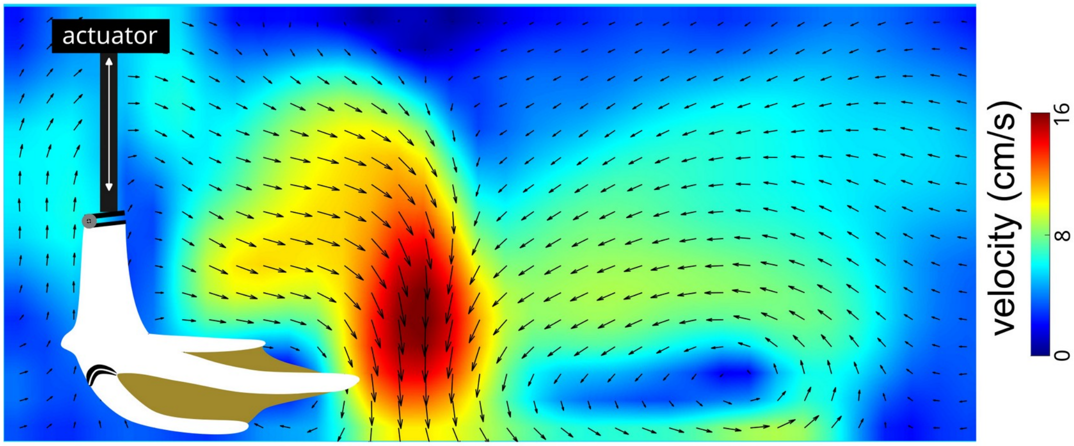

Fig. S7. PIV of morphing foot. Velocity field produced by the mechanical morphing foot.

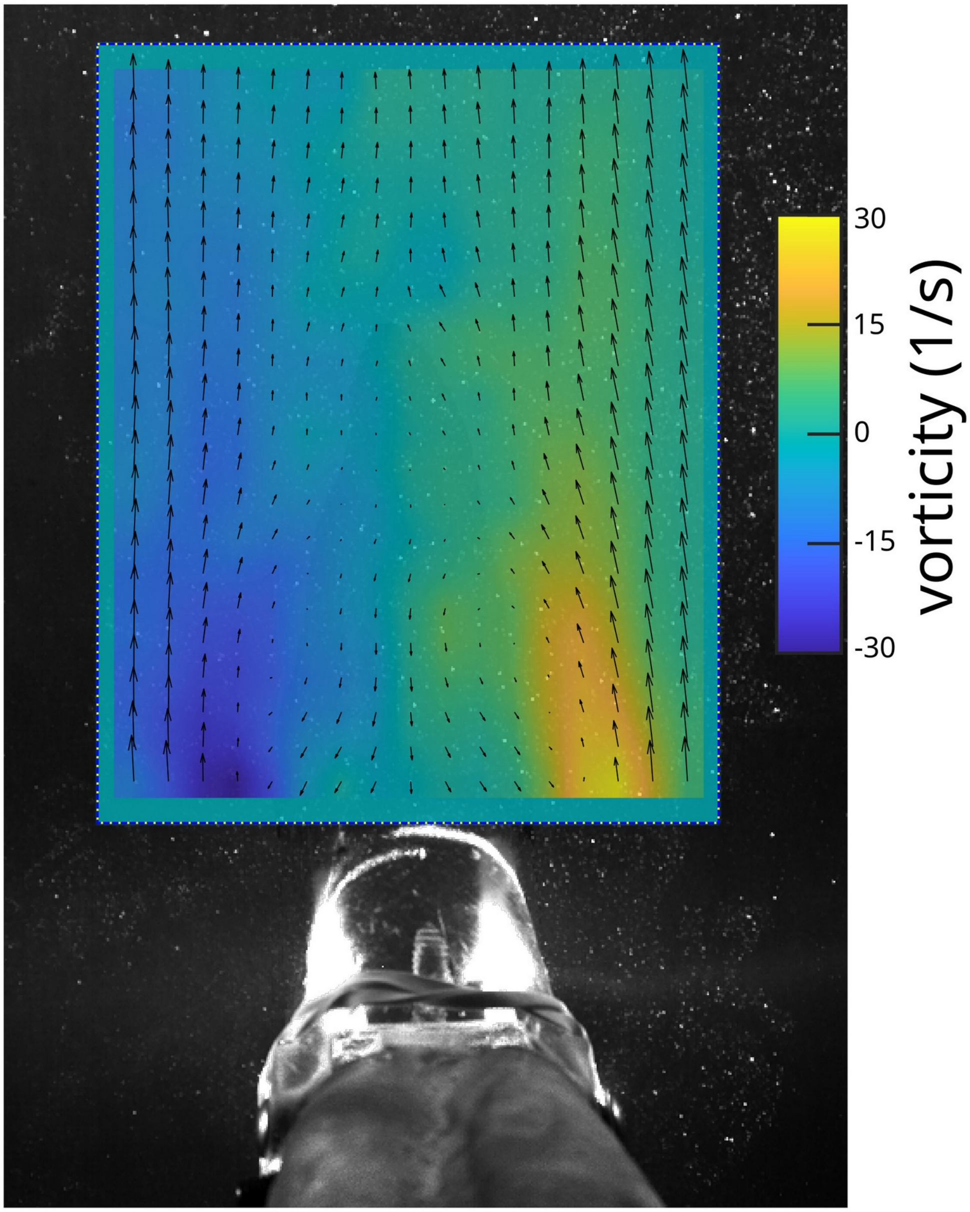

Fig. S8. PIV of a head model during skimming. Mean vorticity field generated by a 3D-printed head during skimming.

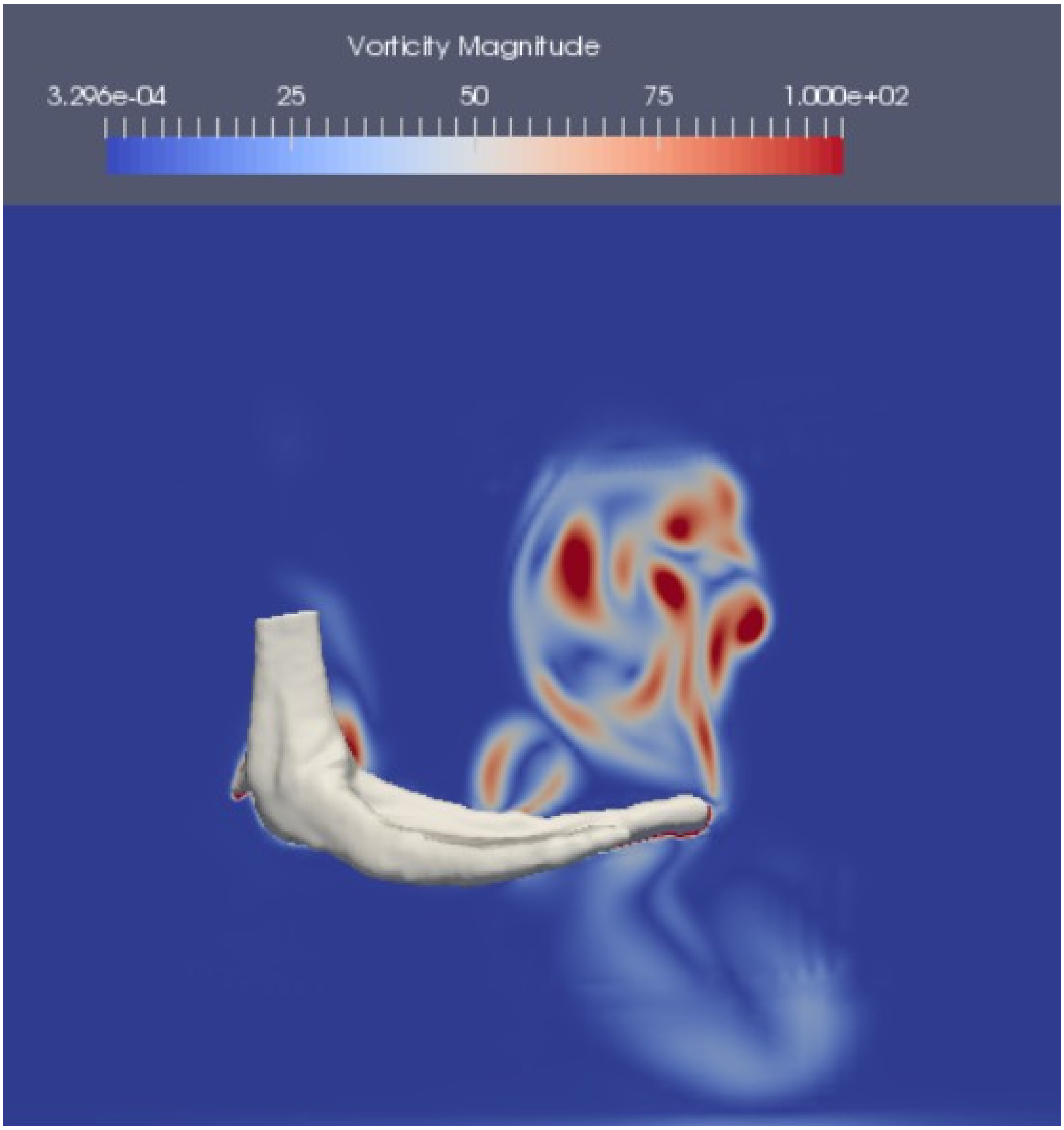

Fig. S9. 3D CFD of a foot during stomping. Vorticity generated by a stomping foot during both downward and upward motion.

Movie S1 (separate file). Tornado-like vortices and head retraction

[00:14 s] Live flamingo generating tornado-like vortices
[00:34 s] 3D-printed beak inducing tornado-like vortices when pulled upward from the bottom

Movie S2 (separate file). Directional flow and beak chattering

[00:14 s] Directional flow produced by a live flamingo during beak chattering
[00:19 s] Flamingo ejecting a water jet along the beak
[00:25 s] Mechanical beak producing directional flow through chattering
[00:40 s] Filtering experiment using a mini pump and a mechanical chattering beak
[00:45 s] Vortices induced by the chattering mandibles and the pump
[00:52 s] No evident vortical structures observed when only the pump is active

Movie S3 (separate file). Horizontal vortex and stomping

[00:14 s] Live flamingos stomping while feeding
[00:37 s] Mechanical morphing foot in motion
[00:46 s] Mechanical morphing foot producing horizontal vortices
[00:54 s] Morphing foot entrapping pond organisms via induced vortices
[01:13 s] 3D computational simulation of vortices generated by a flamingo foot
[01:25 s] Rigid mechanical foot inducing vortices during upward and downward motion

Movie S4 (separate file). Kármán vortex street and skimming

[00:12 s] Live flamingo skimming at the water-air interface
[00:25 s] 3D-printed flamingo head generating Kármán vortices and a recirculation zone
[00:46 s] 3D computational simulation of vortices produced by a flamingo's beak

Movie S5 (separate file). Live brine shrimp (Artemia sp.) and flamingos' vortical traps

[00:20 s] Mechanical beak producing directional motion of live Artemia via chattering
[00:40 s] Capture collection experiment using a mini pump and a mechanical chattering beak
[01:00 s] 3D-printed beak inducing tornado-like vortices of live Artemia when pulled upward.
[01:21 s] Morphing foot entrapping Artemia in an induced vortex
[01:35 s] Artemia collected in the recirculation generated by a printed head during skimming
[02:10 s] Adults and nauplii of brine shrimp
